# The Role of Senolytics in Osteoporosis

**DOI:** 10.3390/biom15081176

**Published:** 2025-08-16

**Authors:** Erman Chen, Jingjing Zhang, Han Chen, Weixu Li

**Affiliations:** 1 Department of Orthopedic Surgery, The Second Affiliated Hospital, Zhejiang University School of Medicine, Hangzhou 310014, China; chenerman@zju.edu.cn (E.C.); 22418465@zju.edu.cn (H.C.); 2 Orthopedics Research Institute, Zhejiang University, Hangzhou 310027, China; 3 Key Laboratory of Motor System Disease Research and Precision Therapy of Zhejiang Province, Hangzhou 310009, China; 4 Clinical Research Center of Motor System Disease of Zhejiang Province, Hangzhou 310009, China; 5 School of Pharmacy, Hangzhou Normal University, Hangzhou 311121, China; 2023112025106@stu.hznu.edu.cn

**Keywords:** cell senescence, clinical trials, flavonoid, osteoporosis, senolytics

## Abstract

Cellular senescence is a fundamental contributor to numerous dysfunctions and degenerative diseases, including osteoporosis. In genetically modified and preclinical animal models, therapeutic strategies targeting persistent senescent cells have been shown to delay and prevent osteoporosis. Senolytics are a class of drugs or compounds designed to selectively eliminate senescent cells without adversely affecting normal cells. In this review, we focus on the role of senolytic agents in regulating bone metabolism and their potential in the treatment of osteoporosis. We discussed major types of senolytics, such as natural compounds, kinase inhibitors, Bcl-2 family inhibitors, inhibitors of the mouse double minute 2/p53 interaction, heat shock protein 90 inhibitors, p53-binding inhibitors, and histone deacetylase inhibitors. This review also highlights the progress of senolytics in clinical trials. However, clinical results diverge from preclinical evidence. Therefore, senolytics should be critically evaluated as a potential therapeutic strategy for osteoporosis, with further validation required.

## 1. Introduction

According to the *World Population Prospects* 2024 report, the global population reached nearly 8.2 billion by mid-2024 and is projected to increase by an additional 2 billion over the next 60 years, reaching approximately 10.3 billion by the mid-2080s [1]. In countries where population growth has already peaked, the proportion of individuals aged 65 years and older is expected to rise rapidly, doubling from 17% in 2024 to 33% by 2054. Aging is the most significant risk factor for numerous chronic diseases, including cardiovascular disease, osteoarthritis, osteoporosis, Alzheimer’s disease, and diabetes [2]. The progressive aging of the global population has consequently positioned osteoporosis as one of the most pressing public health challenges worldwide [3,4].

Osteoporosis is a systemic skeletal disorder characterized by compromised bone strength, predisposing a person to an increased risk of fracture [5,6,7]. In the United States, it is estimated that 10.2 million individuals aged 50 years and older are affected by osteoporosis, whereas an additional 43.4 million have low bone mass, placing them at increased risk [8,9]. Globally, the number of hip fractures was estimated to be approximately 2.7 million in 2010 [10]. In 2019, the incidence and prevalence rates of hip fracture in patients aged 55 years and older were 0.681% and 1.191% [11]. The management of osteoporosis and its associated fractures imposes a substantial burden—both in terms of quality of life and economic cost—on individuals and society at large.

Osteoporosis is characterized by decreased bone mineral density and compromised bone quality, leading to the deterioration of bone microarchitecture and increased bone fragility [6]. Although osteoporosis can occur at any age, it is more commonly observed in postmenopausal women and older men. The etiologies of osteoporosis include decreased hormone levels, genetic predisposition, insufficient calcium and vitamin D intake, lack of exercise, and long-term use of certain medications such as glucocorticoids. Osteoporosis is broadly classified into two categories: primary and secondary. Primary osteoporosis includes postmenopausal osteoporosis, senile osteoporosis, and idiopathic osteoporosis [12].

Bone integrity is maintained through a continuous and spatially coordinated process known as bone remodeling, which involves the coupled activities of bone resorption and bone formation [13]. Alterations in the balance between these two processes are critical determinants of bone mass accrual, maintenance, and loss. Estrogen deficiency is a key pathogenic factor in the development of primary osteoporosis [14]. Aging contributes to the disruption of bone remodeling by promoting immune senescence, a chronic pro-inflammatory state, and increased oxidative stress, all of which play important roles in the pathogenesis of osteoporosis [15,16,17]. More recently, cellular senescence has been identified as an important contributor to osteoporosis, independent of estrogen deficiency [18,19,20].

## 2. Cellular Senescence

Cellular senescence is a process whereby cells gradually lose their proliferative capacity and undergo progressive physiological dysfunction during their lifespan [21]. Various cellular stresses, including DNA damage and oxidative stress, can induce cellular senescence. Senescent cells undergo characteristic morphological alterations, exhibiting reduced intracellular water content, progressive cellular atrophy, imbalanced DNA damage and repair, aberrant protein metabolism, and telomere shortening [22]. Cellular senescence represents a double-edged sword: while it facilitates tissue development and repair, it also plays a pivotal role in various pathological conditions. Skeletal senescence, an integral component of organismal senescence, primarily manifests as progressive physiological degeneration of bone tissue. Following peak bone mass attainment, the aging process leads to progressive bone loss, increased bone fragility, and trabecular microarchitectural deterioration, which are predominantly mediated by the aging of different cells in bone tissues. The accumulation of senescent cells across multiple lineages—including bone marrow mesenchymal stem cells (BMMSCs), osteocytes, osteoblasts, osteoclasts, and adipocytes—contributes to osteoporosis development via diverse cellular mechanisms [23]. Cellular senescence plays a critical role in the pathogenesis of skeletal disorders, with targeted elimination or rejuvenation of senescent cells demonstrating promising therapeutic potential.

The prominent features of cellular senescence are cell cycle arrest and the senescence-associated secretory phenotype (SASP) [24]. Senescent cells typically activate cell cycle inhibitory proteins such as p16 and p21, blocking the transition from the G1 phase to the S phase, ultimately resulting in the loss of proliferative capacity. Senescent BMMSCs can lead to cell cycle arrest, ultimately resulting in the impairment of their osteogenic differentiation potential [25]. SASP comprises a highly complex mixture of secreted cytokines, chemokines, growth factors, and proteases [26]. Its exact composition varies considerably depending on the cellular and tissue context, as well as the senescence-inducing stimulus. These secreted factors mediate communication with neighboring cells and the immune system, thereby influencing the fate of senescent cells. SASP contributes to the pathological processes of osteoporosis through multiple mechanisms. On one hand, the accumulation of senescent cells promotes inflammatory factors such as IL-6, IL-1α, and MMP9, which subsequently activate osteoclastogenesis while simultaneously inhibiting osteoblast differentiation [26]. On the other hand, SASP may induce an imbalance in the adipogenic–osteogenic differentiation of BMMSCs, promoting adipocyte formation while suppressing osteoblastogenesis, thereby contributing to the development of osteoporosis [27]. Studies have reported that NF-κB signaling regulates the SASP and cooperatively activates SASP gene promoters together with the transcription factor C/EBPβ [28]. Inhibition of mTOR reduces IL-1α synthesis, thereby suppressing NF-κB activity and subsequently decreasing SASP [29]. Activation of cGAS stimulates interferon genes (STING) to increase the expression of SASP, thereby promoting senescence [30,31]. During apoptosis, mitochondrial DNA (mtDNA) is released into the cytosol through BAX/BAK-mediated macropore formation. The cytosolic mtDNA subsequently activates the cGAS–STING pathway, ultimately inducing SASP [32].

BMMSCs, multipotent progenitor cells derived from the mesoderm, possess multilineage differentiation potential and are capable of differentiating into adipocytes, osteoblasts, and chondrocytes [33]. The mechanism underlying BMMSC senescence involves alterations in surface markers, differentiation and proliferation capabilities, as well as the influence of oxidative stress. Senescent BMMSCs exhibit a decreased proliferation rate, a tendency to arrest in the G1 phase of the cell cycle, and impaired overall antioxidant capacity [34]. Key signaling pathways, such as p53/p2, p16/RB, TGF-β, Wnt/β-catenin, PI3K/AKT/Mtor, and JAK2/STAT3, critically regulate BMMSCs senescence and dysfunction [35]. FKBP5 mediates β-catenin ubiquitination and degradation, thereby accelerating BMSC senescence, impairing osteogenic differentiation, and contributing to osteoporosis [36]. A high-fat diet promotes BMMSC senescence and osteoporosis through disruption of the VDR–SOD2–ROS axis [37]. In aged BMMSCs, adiponectin receptor 2 (AR2) levels are elevated, while adiponectin receptor 1 levels are decreased. Activation of AR2 suppresses osteogenesis by regulating the MAPK and mTOR signaling pathways [38]. Men1 deficiency induces osteoblast senescence through mTORC1 activation and AMPK inhibition. Osteoblast-specific conditional knockout of Men1 in mice leads to skeletal structural alterations that phenocopy age-related osteoporosis [39].

Emerging studies have increasingly recognized the significance of epigenetic regulation in the etiology and advancement of osteoporosis. Epigenetic modifications are heritable changes in gene expression mediated through DNA methylation, histone modifications, chromatin remodeling, and ncRNAs, independent of sequence alterations [40]. It has been reported that two H3K9 demethylases, KDM3A and KDM4C, regulate heterochromatin reorganization during the senescence of BMMSCs by transcriptionally activating the condensin subunits NCAPD2 and NCAPG2 [41]. Nuclear-receptor-binding SET domain protein 2 improves age-related bone loss by enhancing chromatin accessibility by upregulating histone H3 lysine 36 dimethylation (H3K36me2) and downregulating H3K27 trimethylation (H3K27me3), thereby promoting the expression of osteogenic genes [42]. By erasing H3K9me3 to activate β-catenin/Smad1 transcription, KDM4B therapeutically restores bone–fat equilibrium, offering a strategy against skeletal aging and osteoporosis [43]. These findings suggest that epigenetic rejuvenation may represent a novel strategy for preventing and treating osteoporosis.

## 3. Anti-Osteoporosis Drugs

The main goals in the prevention and treatment of osteoporosis include achieving ideal peak bone mass in adulthood, preventing age-related bone loss, and avoiding falls and fractures. The prevention and treatment of osteoporosis primarily involve three components: foundational measures, pharmacologic interventions, and rehabilitation strategies. Foundational measures include lifestyle modifications, regular physical activity, adequate exposure to sunlight, and nutritional optimization through dietary adjustments. Supplementation with essential nutrients, particularly calcium and vitamin D, is also recommended to support bone health. The higher the daily protein intake of middle-aged and older individuals, the lower the risk of developing osteoporosis [44]. Pharmacological treatments play a critical role in osteoporosis management by increasing bone mineral density, improving bone quality, and significantly reducing the risk of fractures [45]. The drug treatment for osteoporosis should be comprehensively considered based on cause, bone metabolism status, and complications of the patient. The rehabilitation treatments for osteoporosis mainly include exercise therapy and physical therapy. Exercise therapy, such as a resistance-based regimen incorporating weight-bearing exercises, can improve muscle strength and reduce the risk of falls and osteoporotic fractures [46]. Physical therapy, such as pulsed electromagnetic fields and shock waves, can increase bone mass [47,48].

Based on the underlying pathophysiology of osteoporosis, anti-osteoporotic medications can be classified into four categories: bone resorption inhibitors, bone formation stimulators, dual-action agents, and drugs with alternative mechanisms [49,50]. As a chronic disease with a high prevalence and serious consequences, osteoporosis requires the use of multiple drugs for long-term combined or sequential treatment to increase bone density and reduce the risk of fractures.

Bone resorption inhibitors include bisphosphonates, the RANKL monoclonal antibody denosumab, calcitonin, estrogen, and selective estrogen receptor modulators. Bisphosphonates are currently the most widely used anti-osteoporosis drug in clinics. Initial medications for osteoporosis include alendronate sodium or risedronate sodium. If oral medications are not tolerated, zoledronic acid or denosumab can be chosen [51]. Denosumab is a monoclonal antibody that can competitively bind to RANKL, inhibiting the RANK/RANKL signaling pathway, thereby reducing bone resorption and increasing bone density. Calcitonin, estrogen, and selective estrogen receptor modulators are less frequently used clinically than denosumab and bisphosphonates.

Bone formation stimulators primarily consist of parathyroid hormone analogues, such as teriparatide and abaloparatide. Teriparatide, a recombinant human parathyroid hormone analog, stimulates osteoblast activity to promote bone matrix synthesis and bone formation. It is particularly indicated for patients with very low bone mass and very high fracture risk [51]. For patients with an extremely high risk of fractures, teriparatide can be considered for combined treatment with zoledronic acid or denosumab. Compared to teriparatide, abaloparatide demonstrates superior efficacy in reducing fracture and hypercalcemia rates.

Romosozumab, a monoclonal antibody targeting sclerostin, exhibits a dual mechanism of action by simultaneously promoting bone formation and inhibiting bone resorption [52]. Romosozumab demonstrates high efficacy in reducing fracture risk. It is approved for a 12-month course of treatment in osteoporosis. However, romosozumab exhibits a higher incidence of cardiovascular adverse events than alendronate sodium. Therefore, it should not be used in patients with a history of heart attack within one year.

In recent years, researchers have identified several novel mechanisms underlying the pathogenesis of osteoporosis, including alterations in the gut microbiome, dysregulation of iron homeostasis, impaired autophagy, and cellular senescence [53,54,55]. The gut microbiota refers to the collective community of microorganisms inhabiting the human intestinal tract, predominantly bacteria, along with fungi, parasites, viruses, and archaea. Gut microbiota and their metabolites modulate bone metabolism via the gut–bone and gut–brain axes. Osteoporosis is associated with gut dysbiosis and Th17/Treg cell imbalance [56]. Iron homeostasis was defined as the balanced state of iron within an organism, which involves processes including iron uptake, storage, utilization, and excretion. The effect of iron homeostasis on skeletal disorders has been widely explored. Iron overload generates reactive oxygen species (ROS) via the Fenton reaction. These ROS activate multiple intracellular signaling pathways, subsequently promoting bone resorption and suppressing bone formation, thereby contributing to osteoporosis [57]. Autophagy is a fundamental cellular process wherein cells selectively eliminate damaged macromolecules and organelles through lysosomal degradation. Mitophagy-based energy impairment contributes to the pathogenesis of osteoporosis [58]. These emerging insights have led to the exploration of new therapeutic agents for osteoporosis, such as senolytics, alpha-ketoglutarate, probiotics, and hydrogen sulfide [59].

## 4. Senolytics

Cellular senescence plays a critical role in the development and progression of various diseases throughout the human lifespan [60]. With aging, senescent cells accumulate in multiple tissues and organs, disrupting tissue structure and function through the secretion of bioactive factors collectively known as the SASP. Lifestyle interventions, such as diet and exercise, can mitigate the accumulation or detrimental effects of senescent cells by reducing DNA damage, mitochondrial dysfunction, and excessive reactive oxygen species production [61,62]. In addition to these non-pharmacological strategies, four major pharmacological approaches targeting senescent cells are currently under investigation: elimination of existing senescent cells using senolytics, inhibition of the SASP, prevention of senescent cell formation, and reprogramming of senescent cells to restore normal function [63,64].

The expression of Ink4a/Arf is closely associated with tissue aging and disease progression in organs such as the kidneys and testes of rodents [65]. Baker et al. [66] developed a novel transgenic mouse model, INK-ATTAC, which utilizes the aging biomarker p16 (Ink4a) to selectively induce the clearance of p16 (Ink4a)-positive senescent cells upon administration of the synthetic drug AP20187. Treatment with AP20187 in INK-ATTAC mice effectively eliminated p16 (Ink4a)-positive senescent cells and delayed the onset of age-related phenotypes. In aged (24-month-old) INK-ATTAC mice, clearance of p16 (Ink4a)-expressing senescent cells using AP20187 was found to attenuate both trabecular and cortical bone loss, highlighting the role of senescent cells in skeletal aging [67]. However, because genetic strategies such as those used in mouse models are not readily translatable to humans, increasing efforts have focused on developing pharmacological approaches capable of eliminating or neutralizing senescent cells for clinical application.

Senolytics represent a class of agents designed to selectively induce apoptosis in senescent cells, thereby potentially delaying, preventing, or even reversing aspects of the aging process and promoting longevity and health span [68,69]. The first senolytic agents were identified through hypothesis-driven, mechanism-based drug discovery approaches. By analyzing proteomic and transcriptomic datasets, researchers discovered that senescent cells commonly upregulate one or more senescent cell anti-apoptotic pathways (SCAPs). To date, 46 compounds targeting these SCAPs have been identified as possessing potential anti-aging properties [70]. First-generation senolytics, which target various SCAP components, include natural compounds, kinase inhibitors, Bcl-2 family inhibitors or Bcl-2 homology 3 (BH3) mimetics, inhibitors of the mouse double minute 2 (MDM2)/p53 interaction, heat shock protein (HSP)90 inhibitors, p53-binding inhibitors, and histone deacetylase (HDAC) inhibitors [71,72] (Table 1). Advances in high-throughput screening and other technologies have guided the development of second-generation senolytic strategies, such as lysosomal and senescence-associated β-galactosidase-activated prodrugs and nanoparticles [73,74], apoptosis induction via sodium–potassium pump (Na+/K+-ATPase) modulation [75], SASP inhibition [76], and immune-mediated clearance approaches, including chimeric antigen receptor T cells, antibody–drug conjugates, and vaccines [77].

### 4.1. Natural Compounds

#### 4.1.1. Quercetin

Quercetin is a plant-derived flavonoid with diverse biological activities [78]. Its pharmacological effects are broad, encompassing antioxidant and free radical scavenging properties, as well as anti-inflammatory, antiviral, antitumor, hypoglycemic, and immunomodulatory functions [79]. Quercetin promotes osteoblast-mediated bone formation primarily through the Wnt/β-catenin and BMP2/SMADs/runt-related transcription factor 2 (RUNX2) signaling pathways, and by enhancing bone matrix mineralization [103]. Additionally, it inhibits osteoclast-mediated bone resorption via modulation of the OPG/RANKL/RANK and ERK1/2/JNK signaling pathways [104,105,106]. When combined with other bioactive compounds, quercetin exhibits synergistic anti-osteoporotic effects [107]. Numerous studies have demonstrated that the combined treatment of dasatinib and quercetin (D + Q) exerts potent anti-aging effects on non-cancerous cells [108]. Targeting senescent cells with D + Q has been shown to significantly ameliorate postmenopausal osteoporosis, restore mesenchymal stem cell function, promote osteogenic differentiation, and facilitate bone regeneration in osteoporotic models [109]. Our findings suggest that D + Q is a promising therapeutic option for postmenopausal osteoporosis and related disorders. Furthermore, D + Q treatment reduces the senescent cell burden and significantly mitigates radiation-induced bone damage [110]. Therefore, quercetin represents a potential candidate for the prevention and treatment of osteoporosis.

#### 4.1.2. Fisetin

Fisetin is a bioactive flavonoid known for its potent antioxidant and anti-inflammatory properties [80,81]. Similar to other flavonoids, fisetin disrupts the PI3K/AKT signaling pathway, facilitating the elimination of senescent cells [82,83]. It promotes osteogenesis primarily through activation of the GSK-3β/β-catenin signaling pathway, thereby contributing to the prevention of osteoporosis [111,112]. Additionally, fisetin inhibits cell fusion, cytoskeletal remodeling, and bone resorption in RANKL-induced differentiation of mouse macrophages [113]. Fisetin also enhances the deacetylation of peroxisome proliferator-activated receptor gamma (PPARγ) mediated by Sirt1, which suppresses PPARγ transcriptional activity and consequently inhibits adipogenesis [114]. Collectively, these properties position fisetin as a promising candidate for the prevention of osteoporosis.

#### 4.1.3. Piperlongumine

Piperlongumine is a natural alkaloid compound extracted from long peppers, known for its diverse pharmacological activities, including antitumor and antidiabetic effects [84]. It inhibits osteoclast formation and function by suppressing the RANKL-induced p38/JNK-cFos/nuclear factor of activated T cells c1 (NFATc1) signaling pathway, thereby exerting protective effects against osteoporosis [115]. Additionally, piperlongumine inhibits adipogenesis, primarily during the early stages of adipocyte differentiation [116]. These properties suggest that piperlongumine may serve as a potential antiresorptive agent or dietary supplement for the prevention of osteoporosis.

#### 4.1.4. Luteolin

Luteolin is a flavonoid found abundantly in many commonly consumed foods, including broccoli, onions, carrots, peppers, cabbages, and apples [85]. Numerous studies have documented luteolin’s antioxidant and anti-inflammatory properties, as well as its capacity to prevent the onset and progression of various aging-related diseases [86,87]. Luteolin has been shown to inhibit dexamethasone-induced osteoporosis through activation of autophagy [117], and to mitigate osteoblast pyroptosis by stimulating the PI3K/AKT signaling pathway, thereby alleviating postmenopausal osteoporosis [118]. It also significantly suppresses the differentiation of bone marrow mononuclear cells and RAW264.7 cells into osteoclasts [119]. Furthermore, luteolin inhibits 3T3-L1 adipocyte differentiation by reducing reactive oxygen species production [120]. Collectively, these findings suggest that luteolin represents a promising therapeutic strategy for the prevention and treatment of osteoporosis.

#### 4.1.5. Curcumin

Curcumin is a natural yellow polyphenolic compound extracted from the rhizome of *Curcuma longa*, known for its anti-inflammatory, antioxidant, and anticancer properties [88]. It reduces bone tissue inflammation, inhibits osteoclast differentiation and proliferation, promotes osteoblast growth, and mitigates oxidative stress through the coordinated regulation of multiple signaling pathways, including but not limited to the NF-κB, Wnt/β-catenin, PI3K/AKT, and p38 mitogen-activated protein kinase (MAPK) pathways [121]. These multifaceted actions position curcumin as a promising agent for the prevention and treatment of osteoporosis.

### 4.2. Kinase Inhibitors

#### Dasatinib

Dasatinib is an orally active, ATP-competitive dual Src/Bcr-Abl kinase inhibitor with potent antitumor effects [89]. Studies have demonstrated that dasatinib promotes osteoblast differentiation, thereby directly enhancing bone formation. Concurrently, it downregulates RANKL synthesis in osteoblasts, indirectly inhibiting osteoclastogenesis [122]. As previously discussed, numerous studies have shown that the combination of dasatinib and quercetin can delay aging and reduce bone loss. Consequently, dasatinib holds considerable potential as an oral therapeutic agent for the treatment of osteoporosis.

### 4.3. Bcl-2 Family Inhibitors

#### 4.3.1. Navitoclax (ABT-263)

Navitoclax (also known as ABT-263) is a potent inhibitor of Bcl-2 family proteins, including Bcl-xL, Bcl-2, and Bcl-w, and promotes the apoptosis of senescent cells [70]. Oral administration of ABT-263 in mice effectively eliminates aged stem cells, such as bone marrow hematopoietic stem cells and muscle stem cells [90]. By clearing senescent stem cells, ABT-263 exhibits anti-aging effects in human mesenchymal stem cells and may influence bone metabolism [123]. However, despite its promising in vitro anti-aging effects on the skeletal system, evidence suggests that Navitoclax’s efficacy in treating age-related bone loss may be limited and could potentially exert harmful effects [124]. Therefore, further investigation is needed to fully assess the therapeutic potential and safety of Navitoclax for bone loss treatment.

#### 4.3.2. ABT-737

ABT-737 is a BH3 mimetic inhibitor targeting Bcl-xL, Bcl-2, and Bcl-w [91]. It induces apoptosis primarily via the mitochondrial pathway and mitochondrial autophagy [92]. Currently, no studies have investigated the effects of ABT-737 on bone metabolism, and its potential role in the treatment of osteoporosis remains unexplored. Additionally, other Bcl-xL inhibitors with senolytic activity, such as UBX1325, A1331852, and A1155463, have yet to be evaluated for their impact on osteoporosis.

### 4.4. MDM2/p53 Interaction Inhibitors

#### 4.4.1. UBX0101

UBX0101 is a promising small-molecule inhibitor targeting the anti-apoptotic protein p53/MDM2, with the ability to selectively eliminate senescent cells. It has shown potential in preventing or even reversing the progression of osteoarthritis. Animal studies have demonstrated that intra-articular injection of UBX0101 in mice selectively clears senescent cells, inhibits articular cartilage erosion, reduces pain, and decreases the expression of inflammatory markers associated with osteoarthritis [93,94]. However, to date, no studies have investigated the effects or underlying mechanisms of UBX0101 in osteoporosis, warranting further research in this area.

#### 4.4.2. P5091

P5091 is a selective and potent inhibitor of ubiquitin-specific protease 7 (USP7). Its anti-aging effects are partially attributed to the promotion of MDM2 ubiquitination and degradation, which leads to increased p53 levels [95]. Bioinformatics analyses have confirmed that USP7 upregulation is associated with osteoporosis. In vitro studies demonstrate that USP7 positively regulates osteoclast differentiation, whereas treatment with P5091 effectively alleviates bone loss in ovariectomized mice [125]. Additionally, depletion of USP7 and administration of P5091 have been shown to reduce inflammation in senescent bone-marrow-derived macrophages and promote osteogenic differentiation of aged BMMSCs [126]. Furthermore, P5091 enhances early-stage osseointegration, suggesting it may improve the quality of bone integration and have clinical benefits for elderly patients with osteoporosis.

### 4.5. Hsp90 Inhibitors

#### 4.5.1. Geldanamycin

Geldanamycin is a natural inhibitor of HSP90 that specifically disrupts the interaction between the glucocorticoid receptor and HSP90. It has been shown to alleviate acute lung injury and acute respiratory distress syndrome caused by viral infections by modulating the host inflammatory response. In osteoblasts, geldanamycin increases MAPK activation, which in turn upregulates interleukin-6 synthesis induced by extracellular ATP [96]. However, the precise role of geldanamycin in osteoporosis remains to be elucidated.

#### 4.5.2. Tanespimycin

Tanespimycin (also known as 17-AAG) is a potent inhibitor of HSP90 that can induce apoptosis, necrosis, autophagy, and mitophagy in various cell types. Studies have demonstrated that 17-AAG promotes osteoclast differentiation through a mechanism independent of NFATc1, involving an upregulation of microphthalmia-associated transcription factor levels and activity [97]. Furthermore, inhibition of HSP90β by 17-AAG reduces the protein levels of the large tumor suppressor (LATS), leading to LATS inactivation and subsequent activation of Yes-associated protein and transcriptional coactivator with the PDZ-binding motif. This cascade enhances osteogenesis [98]. These findings suggest that tanespimycin holds promise as a therapeutic agent for osteoporosis.

#### 4.5.3. Alvespimycin

Alvespimycin (also known as 17-DMAG) is a potent HSP90 inhibitor characterized by lower hepatotoxicity and greater water solubility compared to geldanamycin. Studies have demonstrated that 17-DMAG promotes the proliferation of terminally differentiated chondrocytes both in vitro and in vivo while preserving their chondrocyte phenotype [99]. However, the specific role of alvespimycin in osteoporosis remains to be elucidated.

### 4.6. p53-Binding Inhibitors

#### FOXO4-DRI

FOXO4-D-retro-inverso peptide (FOXO4-DRI) is a cell-permeable peptide antagonist that disrupts the interaction between FOXO4 and p53. As an anti-aging polypeptide, FOXO4-DRI can selectively induce apoptosis in senescent cells [100]. Chondrocytes pretreated with FOXO4-DRI generate cartilage exhibiting a reduced aging phenotype compared to untreated cells, suggesting its potential in eliminating senescent chondrocytes [101]. Currently, no studies have investigated the effects of FOXO4-DRI on bone metabolism, and its role in osteoporosis remains to be explored.

### 4.7. HDAC Inhibitors

#### Panobinostat

Panobinostat is a novel broad-spectrum HDAC inhibitor that can induce autophagy and apoptosis. It prevents cartilage degeneration by regulating the expression of proteins such as HDAC4, HDAC6, HDAC7, RUNX2, and matrix metalloproteinase-13 [102]. However, the specific role of panobinostat in osteoporosis remains unexplored.

## 5. Clinical Trials

Aging is a process that affects the functions of tissues and organs in all living organisms, leading to changes in life expectancy. One hallmark of aging is the replicative senescence of primary fibroblasts in vitro, which results in an irreversible arrest of the cell cycle. This replicative senescence is primarily triggered by DNA damage caused by telomere shortening [127]. Additionally, other forms of persistent DNA damage—such as those induced by oxidative stress, chemotherapy, oncogenic mitogenic signals, and radiation—can also initiate replicative senescence.

In recent years, eliminating senescent cells to treat various diseases and age-related dysfunctions has garnered considerable attention. This approach is supported by genetic models in which senescent cells are selectively removed by activating pro-apoptotic transgenes driven by p16 (INK4A) and p21 promoters [128,129]. Beyond genetic methods, researchers are actively exploring senolytics as a more feasible strategy for eliminating senescent cells in humans and have conducted some clinical trials (Table 2). Among these, the combination of dasatinib and quercetin is the most extensively studied senolytic therapy [130,131]. This drug cocktail has undergone clinical trials for several diseases, including Alzheimer’s disease, idiopathic pulmonary fibrosis, liver fibrosis, and chronic kidney disease [132,133,134]. A clinical trial (ClinicalTrials.gov ID: NCT04313634) conducted by the Mayo Clinic demonstrated that levels of procollagen type I N-terminal propeptide (PINP) were higher in the dasatinib and quercetin treatment group at weeks 2 and 4 compared to controls, although no significant differences were observed by week 20 [135]. However, the potential side effects of senolytics restrict clinical application. The most common side effect of Navitoclax was thrombocytopenia [136,137]. Quercetin may enhance the nephrotoxic effect or promote tumor development [138]. Overdose of dasatinib treatment may cause gastrointestinal bleeding [139]. The common side effects of dasatinib include gum bleeding, oral ulcers, stomach pain, and numbness in limbs. During the medication period of dasatinib, regular tests of the liver and kidneys are required.

While the combination of D + Q reduces senescent cell burden across multiple cell types and demonstrates anti-aging effects, including osteoporosis prevention in animal models, clinical trial data from postmenopausal osteoporotic women revealed that intermittent D + Q administration failed to suppress bone resorption [135]. Similarly, although fisetin shows potent anti-osteoporotic effects in both in vitro and in vivo studies, the same clinical trial demonstrated that intermittent fisetin treatment unexpectedly increased serum C-terminal telopeptide (a bone resorption marker) at week 20 while reducing P1NP (a bone formation marker) at week 2, with no significant improvement in hip and lumbar bone mineral density. These paradoxical findings highlight the substantial translational gap between preclinical anti-aging research and clinical applications, warranting further investigation to elucidate the underlying mechanisms. Interestingly, unlike other senolytic agents, navitoclax does not inhibit osteoporosis but rather induces bone loss [124]. Upon closer examination of the experimental data, we found that the study used navitoclax at 5 μM, a concentration that markedly promotes apoptosis of BMMSCs and increases cytotoxicity. This contrasts sharply with navitoclax’s typical concentration for 50% of maximal effect values (<0.1 μM) in other cell lines such as NCI-H146 and NCI-H1417 [140,141]. These findings suggest the reported bone loss effects may be concentration-dependent, warranting further investigation of the skeletal effects of lower navitoclax concentrations. Furthermore, current preclinical research on other senolytics remains limited, with their precise mechanisms and molecular targets yet to be fully elucidated. As research advances in this field, we anticipate that subsequent clinical trials will be initiated to evaluate their therapeutic potential.

## 6. Conclusions

Currently, research on senolytics and cellular senescence has become quite extensive, with a growing number of studies focusing on bone metabolism and osteoporosis. However, clinical research and translational applications of senolytics for osteoporosis remain limited. The inconsistencies between preclinical studies and clinical trial outcomes have further limited the potential applications of senolytics in humans. More comprehensive research is warranted to investigate the effects of senolytics on bone metabolism and osteoporosis prevention, including optimal dosing, treatment frequency, and timing of administration. Clinical institutions should prioritize multicenter randomized controlled trials and other high-level clinical studies to elucidate the precise therapeutic benefits of senolytics for osteoporosis.

To date, most studies have concentrated on the dasatinib and quercetin cocktail as well as fisetin. While select senolytics exhibit robust pro-longevity and osteoprotective effects preclinically, their translational potential for osteoporosis remains unproven. Therefore, senolytics should be viewed as a potential therapeutic strategy for osteoporosis with cautious optimism, and further studies are warranted to validate their clinical potential.

## Figures and Tables

**Table 1:** Identified senolytic agents.

Category	Agent	References
Natural compounds	Quercetin	[78,79]
	Fisetin	[80,81,82,83]
	Piperlongumine	[84]
	Luteolin	[85,86,87]
	Curcumin	[88]
Kinase inhibitors	Dasatinib	[89]
Bcl-2 family inhibitors	Navitoclax (ABT-263)	[90]
	ABT-737	[91,92]
MDM2/p53 interaction inhibitors	UBX0101	[93,94]
	P5091	[95]
Hsp90 inhibitors	Geldanamycin	[96]
	Tanespimycin	[97,98]
	Alvespimycin	[99]
p53-binding inhibitors	FOXO4-DRI	[100,101]
HDAC inhibitors EYA1	Panobinostat	[102]

**Table 2:** Senolytics in clinical trials.

Senolytic Compound	Targeted Pathology	ClinicalTrials.gov ID
Quercetin	Bone Health and Markers	NCT05371340
Quercetin and Dasatinib	Osteoporosis	NCT06018467
Fisetin	Osteoarthritis	NCT04815902
Luteolin	Osteoarthritis	NCT04638387
UBX0101	Osteoarthritis	NCT04229225, NCT03513016, NCT04349956

## Data Availability

No new data were created.
